# Role of nitric oxide in management of acute respiratory distress syndrome

**DOI:** 10.4103/1817-1737.41914

**Published:** 2008

**Authors:** A. H. Akmal, Mohd Hasan

**Affiliations:** *Intensive Care Unit, Department of Intensive Care Medicine, Salmaniya Medical Complex, Manama, Bahrain*

**Keywords:** Acute respiratory distress syndrome, nitric oxide, PAC, ventilator

## Abstract

The current mortality rate of patients suffering from acute respiratory distress syndrome (ARDS) is between 45% and 92%, with most dying within the first two weeks of the illness. In an effort to combat such an alarmingly high mortality rate, various treatment therapies such as low tidal volume ventilation strategies, corticosteroid therapy, and use of nitric oxide (NO) have been attempted in the management of patients with ARDS. Three cases which were admitted to the ICU and confirmed to have ARDS were unable to be weaned from ventilatory support, and nitric oxide therapy was initiated. It improved patients' oxygenation for short periods of time but did not affect the mortality. The patients could not be weaned from the ventilator and expired.

## Introduction

The extraordinary mortality rate of patients suffering from acute respiratory distress syndrome (ARDS) is between 45% and 92%, with most dying within the first two weeks of the illness.[1] In an effort to combat these alarmingly high mortality rates, various treatment therapies, including low tidal volume ventilation strategies,[2,3] corticosteroid therapy,[4,5] and use of nitric oxide, for ARDS patients[6] have been advocated.

ARDS, an acute lung injury in its aggravated form, is characterized by a sudden, mostly generalized inflammation of the lung, which, in the further course, induces (1) noncardiogenic pulmonary edema, (2) pulmonary arterial hypertension, (3) reduction of total compliance of the lung, and (4) progressive systemic hypoxemia due to a pulmonary ventilation / perfusion mismatching, leading to an increased intrapulmonary right-to-left shunt area. Pulmonary hypertension causes a rise of the microvascular filtration pressure in the lung and therefore, development of an interstitial pulmonary edema, as well as excessive stress and dysfunction of the right ventricle.

There are four criteria to diagnose ARDS:

1. Acute onset
2. Bilateral infiltrates on frontal chest radiograph
3. Hypoxemia (PaO_2_/FiO2 ratio < 200 mm Hg)
4. Pulmonary capillary wedged pressure < 18 mm Hg[7]

Until recently, the only therapeutic means of reestablishing a safe level of oxygenation in patients with ARDS was mechanical ventilation with high inspiratory oxygen concentrations (fraction of inspired oxygen [FiO2]) and positive end-expiratory pressure (PEEP). Such an approach could be counterproductive in some patients because high O_2_ concentration and high airway pressure can cause further lung damage due to O_2_ toxicity and pulmonary barotrauma. A pharmacologic approach is emerging as an adjunct to conventional management, and it aims to decrease the need for high O_2_ concentrations and high airway pressures. The objective of most of these newer modalities is to redistribute the pulmonary blood flow preferentially toward well-ventilated alveoli.

Ventilation/perfusion mismatch is one of the hallmarks of ARDS. In the lung, regions of normal ventilation-perfusion ratio (V/Q) coexist with regions of low V/Q. A large proportion of the lung which appears ventilated on chest radiograph and computerized thoracic scan is poorly perfused or not perfused at all (alveolar dead space). At the same time, parts of the nonventilated zones continue to be perfused due to the failure of hypoxic pulmonary vasoconstriction (HPV), a reflex which tends to limit the pulmonary blood flow perfusing poorly oxygenated alveolar spaces. High tidal volumes (V_T_) and, consequently, high airway pressures are needed to maintain normal arterial carbon dioxide tension (PaCO_2_) in the face of increased alveolar dead space.

Acute pulmonary hypertension, which is frequently observed in patients with ARDS, is a result of pulmonary vasoconstriction characterizing the early stages of ARDS, and of the remodeling of the pulmonary vasculature that is observed in the late stages of ARDS. This pulmonary vasoconstriction can, in turn, be due to the HPV reflex or chemical mediators such as thromboxane A_2_ and platelet activating factor. Anatomical remodeling consists of muscular hypertrophy, microthrombosis, fibrosis, and destruction of pulmonary vessels.

Theoretically, selective constriction of the pulmonary vessels in the nonventilated zones or selective vasodilation in the ventilated zones should decrease the V/Q mismatch. Administration of selective pulmonary vasoconstrictors and vasodilators is the basis of the pharmacologic approach to hypoxemia during ARDS. Ideally, these vasoactive drugs act on the pulmonary circulation while having little or no effect on systemic circulation.

## Case 1

A 62-year-old man was admitted to the hospital with complaint of fever and abdominal pain of 2 days' duration. The pain was mainly in the right hypochondriac region and epigastrium, and it radiated to the back and was associated with vomiting. The patient had hypotension and was shifted to the intensive care unit (ICU) for further management.

**Figure 1:**
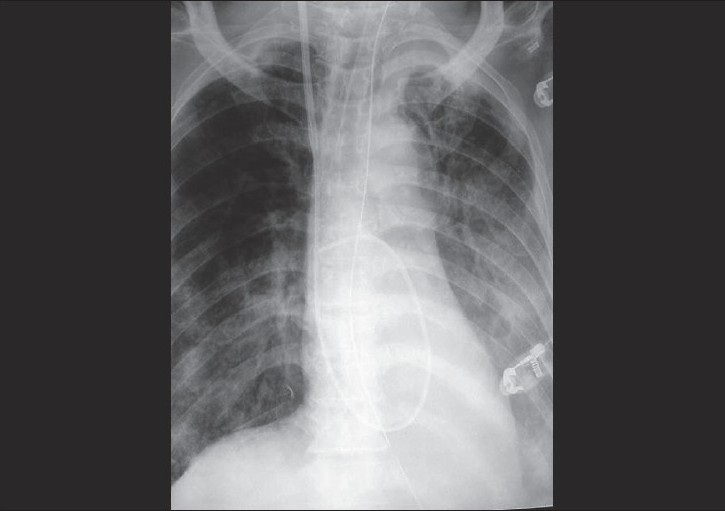
Chest radiograph showing bilateral infiltrates with pulmonary artery catheter

On examination, the patient was tachycardic, febrile, with rigid abdomen, transient hypocalcemia, and tachypnea. Chest radiograph showed left pleural effusion with atelectasis; ultrasound of the abdomen showed gallstones with pancreatic enlargement; CT of the abdomen showed necrotic pancreatic tissue. Laboratory results showed a total white cell count (WBC) of 24 × 10^9^/L and serum amylase of 644 IU. The patient was diagnosed to have acute pancreatitis and was resuscitated with fluids and oxygenation. On the second day, he desaturated; he was intubated and supported with mechanical ventilation. Chest radiograph showed bilateral diffuse infiltrates [Figure 1]. A Swan-Ganz catheter (pulmonary artery catheter, PAC) was inserted, which showed a pulmonary capillary wedge pressure (PCWP) of 13 mmHg and a PaO_2_/FiO_2_ ratio < 200 mm Hg; and the patient was diagnosed to have ARDS secondary to acute pancreatitis. Low tidal volume strategies (6 mL/kg) with high PEEP were applied, and later the patient went into acute renal failure and required hemodialysis.

The patient kept on desaturating. He required very high PEEP and high O_2_ consumption, and his oxygen saturation (SaO_2_) was maintained in the range of 85% to 87%. Later the patient was put on pressure-controlled mechanical ventilation (PCV) with inverse ratio but did not show any improvement in oxygenation. The nitric oxide therapy protocol was initiated; and on the second day, the patient started to show improvement in his oxygenation. The FiO_2_ was reduced from 90% to 50% and the PEEP reduced from 16 to 7 cm H_2_O. He was continued on NO for 13 days and again desaturated, and he required a higher PEEP and higher FiO_2_. His general condition deteriorated still further and he expired.

## Case 2

A 28-year-old lady, a known case of epilepsy maintained on Lamotrigine, was admitted to the ICU after a 3-day history of continuous high-grade fever, cough, mild hemoptysis, nausea, vomiting, and diarrhea. Initially she was tachypneic (RR, 50/min), tachycardic (HR, 160/min), normotensive (BP, 160/86 mmHg) and febrile (temperature, 40°C), and with bilateral coarse crepitations in her chest. Chest radiograph showed bilateral lung infiltrations. Arterial blood gases revealed hypoxemia (PaO_2_, 50 mm Hg) with a PaO_2_/FiO2 ratio of 127 mm Hg. Echocardiography was done, which excluded left atrial hypertension.

Accordingly, the patient was diagnosed as ARDS secondary to community-acquired pneumonia. She was maintained with mechanical ventilation, in a pressure-controlled mode. Antibiotics, activated protein C, and maximum inotropic support were given to the patient as her organs started to fail. She required a high level of FiO_2_ (90%-100%) in order to keep oxygen saturation ≥ 90%. Her saturation was fluctuating, for which oxygen insufflations, alveolar recruitment, and prone position were all tried for the first two days of her ICU stay, with no improvement. Nitric oxide therapy was started on the second day at a rate of 5 to 10 ppm. Her saturation improved, mainly on the next day of nitric oxide therapy, and it was possible to reduce the FiO_2_ to 60%. Unfortunately this effect was lost after 4 days, when her general condition deteriorated and she went into cardiac arrest.

## Case 3

A 69-year-old man was admitted through the Accident and Emergency Department with shortness of breath, cough associated with fever, and right-sided chest pain for the past 3 days. On arrival to the ICU, the patient was sick looking, hypotensive, tachycardic; and his oxygen saturation was 88% on 15L O_2_ by facemask. His WBC was 23 × 10^9^/L with 10% band forms, and chest radiograph showed right mid-zone pneumonia. The patient was started on antibiotics and other supportive therapy; and on the second day, his condition deteriorated and he was intubated and placed on mechanical ventilation. His chest radiograph showed diffuse bilateral infiltrates, and FiO_2_/PaO_2_ ratio was < 200 mm Hg; he was diagnosed as ARDS.

Protective lung strategies were applied with low tidal volume and high PEEP, but he was requiring very high FiO_2_, > 80%, to maintain SaO_2_ > 88%. Later the patient deteriorated further; and on the third day, NO was started as per protocol and after 10 to 12 hours of NO, the patient started showing improvement in his oxygenation; his SaO_2_ improved and he required a FiO_2_ < 45% to maintain SaO_2_ > 90%. Nitric oxide was continued for 6 days, and the patient was requiring very low FiO_2_ to maintain SaO_2_ > 90%. On day 7 of NO therapy, the patient started deteriorating and again required very high FiO_2_, but he did not recover and expired.

## Discussion

Nitric oxide is a lipophilic gaseous molecule that readily diffuses across pulmonary membranes, causing localized vasodilatory effects in the pulmonary vascular bed. It is used to combat vasoconstriction, V / Q mismatching, arterial hypoxemia, and pulmonary hypertension associated with ARDS. Nitric oxide has beneficial effects in the lungs as its vasodilatory actions are limited to the pulmonary vasculature. This is due to the inactivation of NO as it reacts with hemoglobin (producing methemoglobin) and oxygen dissolved in plasma. Therefore, its systemic hemodynamic effects are negated. More than 30 studies on the use of NO for ARDS in humans have been completed, but the majority of them are small and fraught with flaws and limitations.[8]

Nitric oxide is a multipotent endogenous messenger molecule that is extensively involved in the regulation of vascular tone. Nitric oxide is also a well-known environmental pollutant, emanating from internal combustion engines and factories. Nitric oxide is toxic in higher concentrations, and the legal limit for NO occupational exposure is 25 parts per million (ppm). However, NO gas mixed in nitrogen can be used for medical purposes as an inhalable drug, administered together with the inspiratory gas. Nitric oxide passes through the alveolar membrane and reaches the blood in the pulmonary vasculature, where it is taken up and possibly absorbed by hemoglobin before it reaches the systemic circulation. Inhaled nitric oxide (INO) is considered a selective pulmonary vasodilator because it dilates the pulmonary vasculature that is in contact with ventilated alveoli, while having no effect on the resistance of the systemic vasculature.[9]

Since NO exists in a gaseous form, it can be applied to the pulmonary vessels by administering it as an inhaled gas. What this means is that when NO is inhaled, it selectively dilates blood vessels in only those lung segments that are actively participating in gas exchange (oxygen and carbon dioxide) at the alveolar-capillary level. In other words, this increases the blood flow to areas of the lung where oxygen is being provided and thus improves oxygen levels in the body. This is known as ventilation / perfusion (V / Q) matching.

After the NO is inhaled and passes through the lungs and into the patient's blood stream, its effects are quickly deactivated. This is because NO quickly reacts with the iron-containing pigment (hemoglobin) of the red blood cell that functions to carry oxygen from the lungs to the tissues. Hemoglobin inactivates NO; and thus when it is carried to the rest of the body, it does not cause vasodilation to blood vessels beyond the lung area. This is in stark contrast with some of the other pulmonary vasodilator drugs that not only cause vasodilation of blood vessels in and around the lungs, but also cause vasodilation throughout the body. This can potentially lead to a serious decrease in a patient's blood pressure.

Virtually since the discovery of NO for medical use in the mid-to-late '80s, it has been tried on patients with ARDS in many trials. Numerous formal studies have been completed that examined the effect NO had on ARDS patients. Virtually every study found that inhaled NO 1) induces a redistribution of blood flow in the lungs to areas that are well ventilated, 2) reduces the blood pressure in the arteries surrounding the lungs, and 3) improves oxygen levels in the blood. Studies also showed that not every patient responds to inhaled NO in the same manner. Some patients have an almost immediate positive and recognizable response, while others have a limited response. Some studies have found that only about one third of the patients with ARDS due to sepsis had a positive response to inhaled NO. Among other factors, patients who had high blood pressure in the arteries near the lungs and who demonstrated a positive response to PEEP (positive end-expiratory pressure from the ventilator) appeared to be most likely to have a positive response to inhaled NO. For some patients, the positive response to inhaled NO appears to last for only hours to days, while others respond positively for weeks. The reason for this phenomenon is still being investigated.[10]

Promising results are reported with NO for the treatment of ARDS. This gas produces localized pulmonary vasodilatation that results in increased arterial oxygenation (PaO_2_), decreased shunt fraction, and decreased pulmonary resistance, without affecting systemic hemodynamics.[11,12] A wide range of doses (0.01-100 ppm) were administered, but without a clear dose-response relationship. Most trials reported that doses less than 40 ppm and higher doses (36 or 40 ppm vs .18 or 20 ppm) did not provide added benefit in most patients.[13]

Marked variations in the effects of NO may be due to different preexisting pulmonary diseases, as well as continuing infusions of vasoactive drugs. Differences in gas mixing systems, use of extracorporeal lung support (ECLS), dose, duration of therapy, and natural history of a patient's illness may also contribute to an inconsistent response.[14,15] In some small noncomparative trials in patients with ARDS, NO improved arterial oxygenation in most patients, as shown by an increase in the PaO_2_:FiO_2_ ratio and decreased pulmonary artery pressures without compromising systemic hemodynamics or causing serious side effects.[16–18] Before the advent of inhaled NO, prostacyclin, an intravenous vasodilator, had been studied for ARDS management. This agent is of limited benefit since it can reduce mean arterial pressure (MAP), increase intrapulmonary shunting, and lead to reduced oxygenation. Unlike intravenous prostacyclin, inhaled NO does not reduce systemic arterial pressure. Localized redistribution of pulmonary blood flow as a result of reversed regional vasoconstriction in ventilated lung regions is the most logical explanation for NO's efficacy.[19,20]

The most important goals in the treatment of ARDS are to reduce mortality and improve patient outcomes (e.g., decrease long-term disability). Trials conducted to date have focused primarily on gas exchange and hemodynamics. Other values may be important indicators of prognosis, such as epithelial and endothelial barrier function, accumulation of lung edema, inflammatory events, host-defense competence, and fibrotic processes.[21]

The multiple studies suggested good outcome of NO therapy in ARDS; however, our reported patients did not benefit long term except for some improvement in oxygenation for short periods, but it was not possible to wean the patient from the ventilator. In our experience, NO improves oxygenation for a short period but does not improve mortality. Further studies should assess these measures and focus on impact of NO therapy on morbidity and mortality.

## Conclusion

The most profound effects of NO when used in the management of ARDS generally occur within the first few days following the commencement of therapy, and do not necessarily improve significantly with time. The effects of decreasing pulmonary artery pressure (PAP) and improving oxygen saturation (SaO_2_) have been proven in several studies.[1–3] In patients that respond to the NO therapy during the first day, the FiO_2_ can be decreased and the pitch of ventilatory support can be reduced over the following days.

The difficulty in analyzing the success or failure of patient outcomes (mortality and/or length of stay) for patients with ARDS is that the reversal of the lung disease may be obscured by the development of other organ system malfunctions that all too often occur with ARDS. Since NO is a relatively new treatment modality,[1,6,8–11] there have been only a limited number of studies that have reported patient outcome. Most published research has focused on the physiological benefits and improved cardiopulmonary conditions in response to the inhaled NO.
